# Supplementary material for: Tridimensional visualization reveals direct communication between the embryo and glands critical for implantation
Source: Nat Commun. 2018 Feb 9;9:603. doi: 10.1038/s41467-018-03092-4 (PMC5807548; doi:10.1038/s41467-018-03092-4)
Supplement: Supplementary file 1 — Supplementary Information [file 41467_2018_3092_MOESM1_ESM.pdf]

Tridimensional visualization reveals direct communication between the embryo and glands critical for implantation

Jia et al.

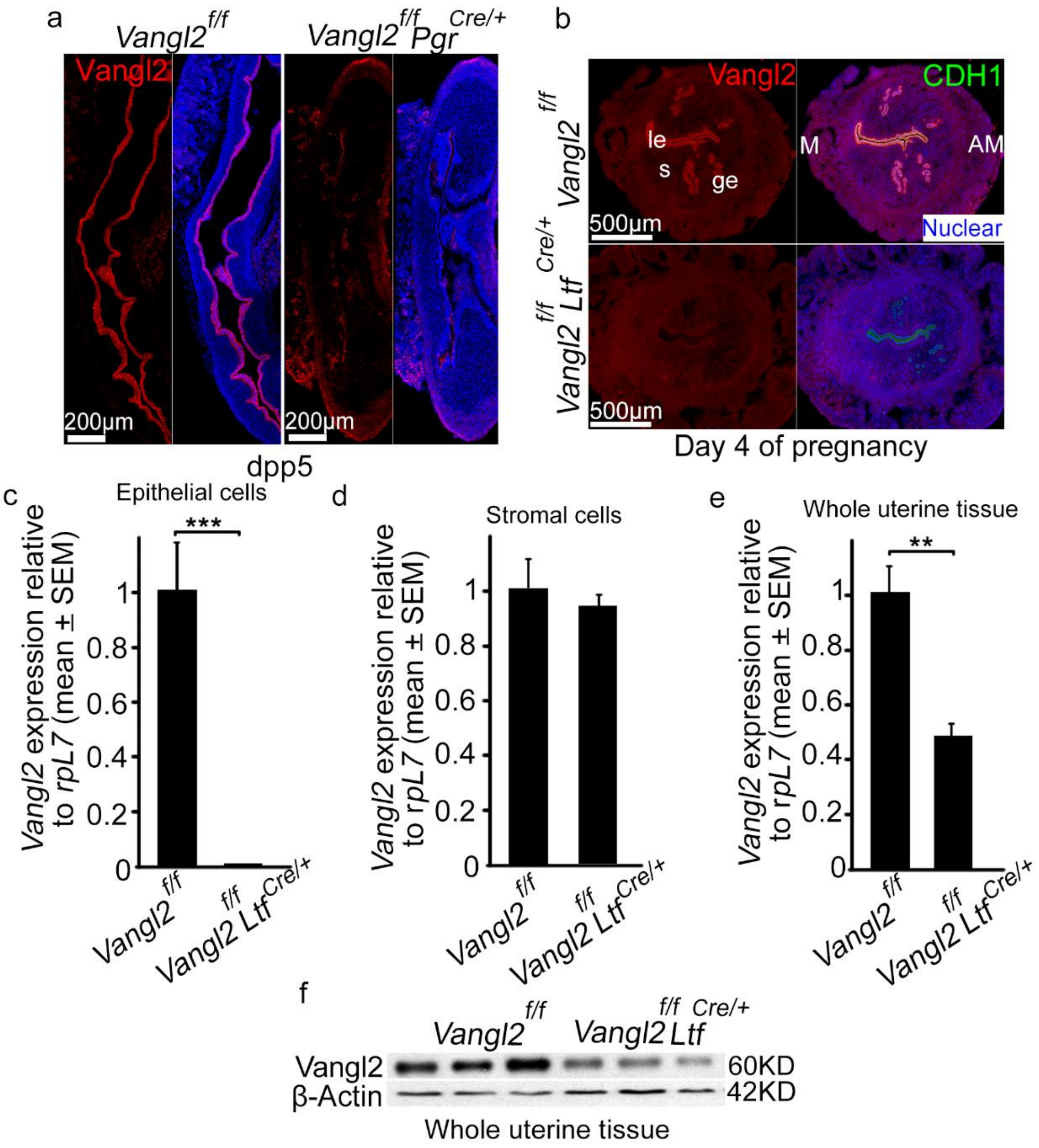

Supplementary Figure. 1

Supplementary Figure 1 | Efficiency of *Vangl2* deletion in *Vangl2*$^{f/f}$*Ltf*$^{Cre/+}$ mice. a, IF of Vangl2 in dpp5 (postpartum day 5) uterus of *Vangl2*$^{f/f}$ and *Vangl2*$^{f/f}$*Pgr*$^{Cre/+}$ mice (n = 3 independent experiments). Scale bar: 200 μm. **b,** IF of Vangl2 and CDH1 in day 4 pregnant uteri of *Vangl2*$^{f/f}$ and *Vangl2*$^{f/f}$*Ltf*$^{Cre/+}$ mice (n = 3 independent experiments). ge, glandular epithelium; le, luminal epithelium; s, stroma; M, mesometrial pole; AM, antimesometrial pole. Scale bar: 500 μm. **c,**

qRT-PCR of *Vangl2* in separated epithelial cells of *Vangl2*$^{f/f}$ and *Vangl2*$^{f/f}$*Ltf*$^{Cre/+}$ mice on day 4 (Mean ± s.e.m. is derived from the indicated number of samples and analyzed by Student's *t* test, ***p < 0.001). **d,** qRT-PCR of *Vangl2* in day 4 separated stromal cells of *Vangl2*$^{f/f}$ and *Vangl2*$^{f/f}$*Ltf*$^{Cre/+}$ mice (Mean ± s.e.m. is derived from the indicated number of samples and analyzed by Student's *t* test). **e,** qRT-PCR of *Vangl2* in whole uteri of *Vangl2*$^{f/f}$ and *Vangl2*$^{f/f}$*Ltf*$^{Cre/+}$ mice on day 4. (Mean ± s.e.m. is derived from the indicated number of samples and analyzed by Student's *t* test, **p < 0.01). Data are shown as mean ± SEM (n=4). **f,** Western blotting results of Vangl2 in whole uterine tissues of *Vangl2*$^{f/f}$ and *Vangl2*$^{f/f}$*Ltf*$^{Cre/+}$ mice (n=3).

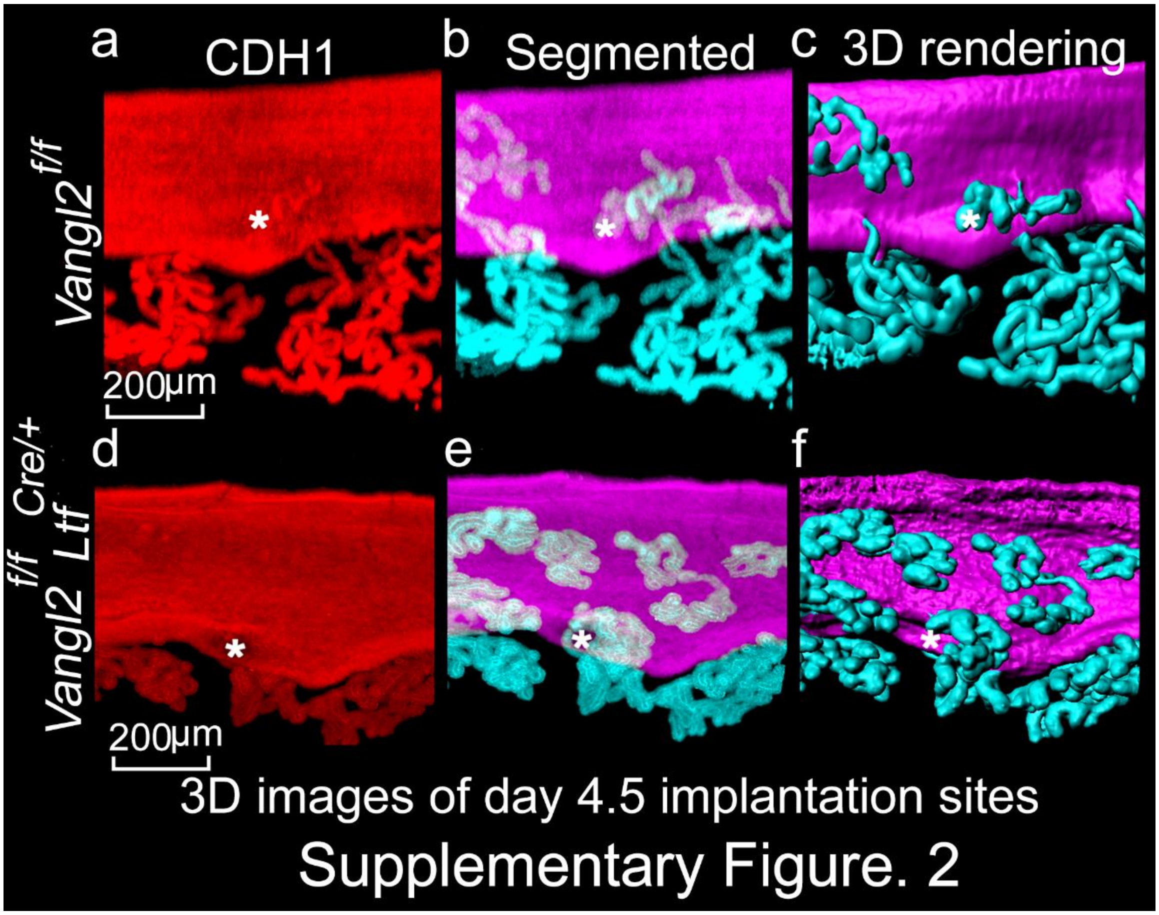

Supplementary Figure 2 | 3D imaging of day 4.5 implantation sites in *Vangl2*$^{f/f}$ and *Vangl2*$^{f/f}$*Ltf*$^{Cre/+}$ mice. a-f, Images of CDH1 immunostaining, segmented and 3D rendering in day 4.5 *Vangl2*$^{f/f}$ and *Vangl2*$^{f/f}$*Ltf*$^{Cre/+}$ implantation sites (n = 3 independent experiments). Images are generated by Nikon A1R Multiphoton with LWD 16X water objective with 3 μm Z-stack. *, location of embryos. Scale bar: 200 μm.

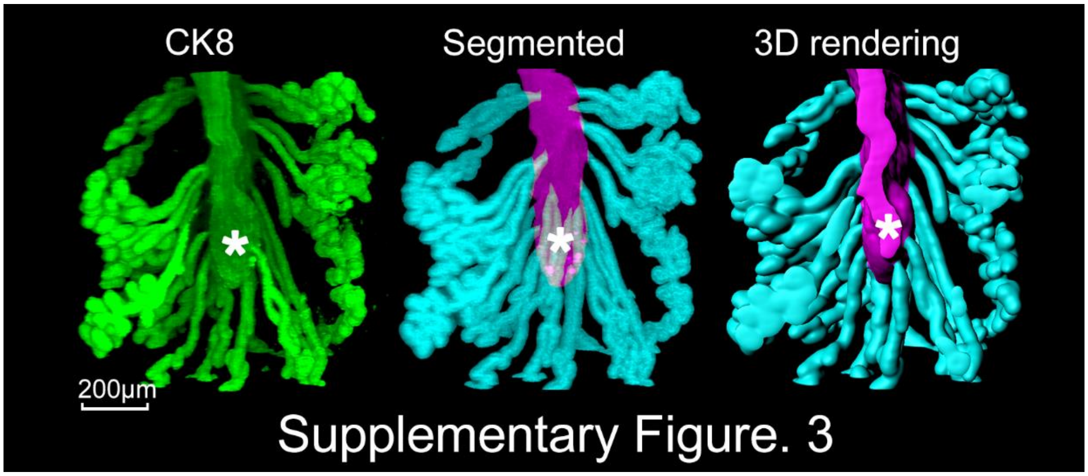

Supplementary Figure 3 | 3D imaging of day 5 implantation sites in *Vangl2*$^{f/f}$ mice staining with CK8 at sectional view. Images of CK8 immunostaining, segmented and 3D rendering of a day 5 implantation site generated by two-photon microscopy (n = 3 independent experiments). *: crypt containing the embryo. Scale bar: 200 µm.

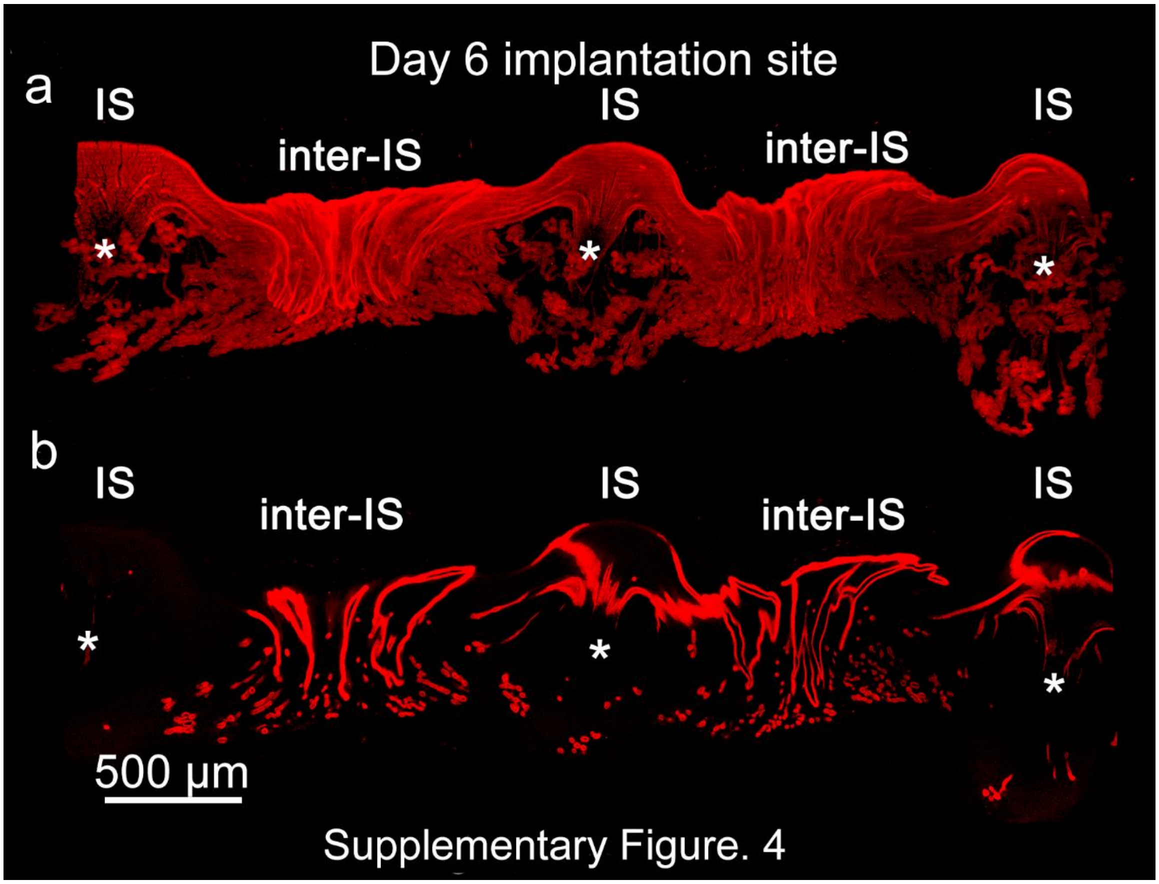

Supplementary Figure 4 l Images of a day 6 uterus with implantation sites and inter-implantation sites stained with CDH1. a, 3D image of D6 with three implantation sites and two inter-implantation sites (n = 3 independent experiments). Note glands at the inter-implantation sites are compressed, while glands at implantation sites are extended and developed. **b,** A sectional view of 3D image. IS, Implantation site; inter-IS, inter-implantation site. Images were generated by Nikon A1R Multiphoton Microscope; Plan Apo λ 10X objective with 12 µm Z-stack. *, location of embryos. Scale bar: 500 µm.

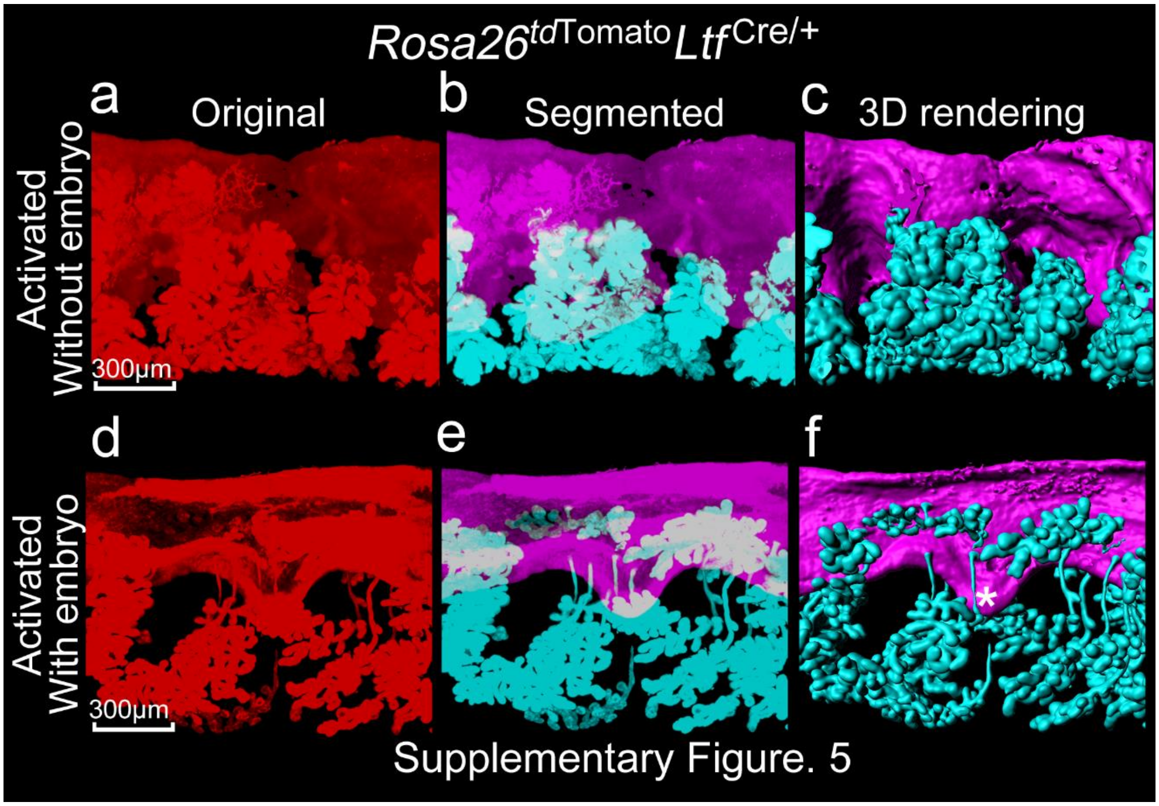

Supplementary Figure 5 l Images of activated uterus with or without implantation sites in the same mouse. a-c, shows 3D image of one uterine horn after termination of delay with estrogen in the absence of blastocys in *Rosa26*^{tdTomato}*Ltf*$^{\mathrm{Cre/+}}$ reporter mouse (n = 3 independent experiments). **d-f,** Images after termination of delay with estrogen in the presence of blastocyst from the contralateral horn of the same reporter mouse (n = 3 independent experiments). (Images were generated by Nikon A1R Multiphoton Microscope; LWD 16X water objective with 3 µm Z-stack). *location of embryos. Scale bar: 300 µm.

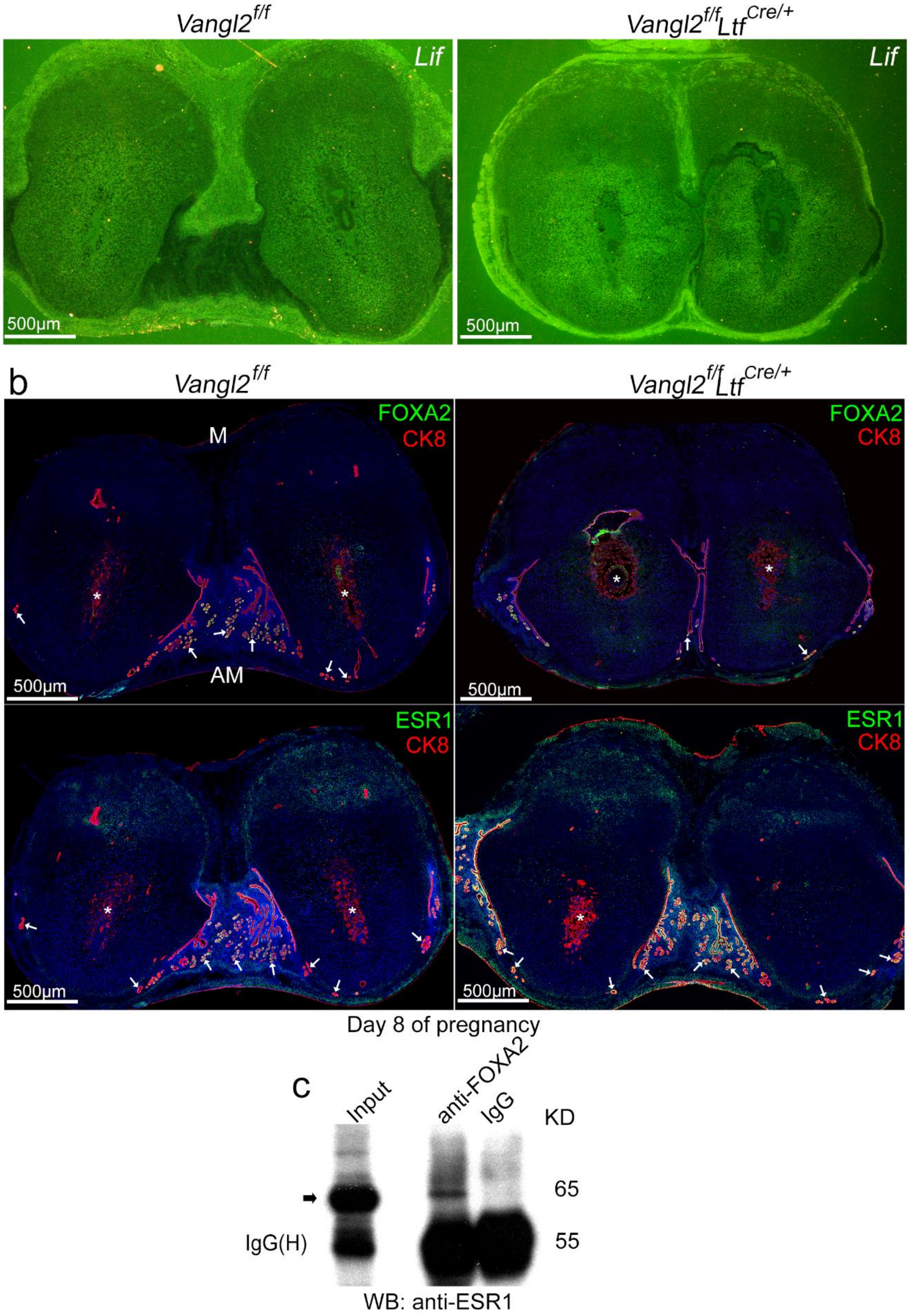

Supplementary Figure. 6

Supplementary Figure 6 l a, *In situ* hybridization of *Lif* in day 8 uteri of *Vangl2*$^{f/f}$ and *Vangl2*$^{f/f}$*Ltf*$^{Cre/+}$ mice (n = 3 independent experiments). Scale bar: 500 μm. **b,** Co-staining of FOXA2 and CK8, ESR1 and CK8 in longitudinal sections of day 8 implantation sites in *Vangl2*$^{f/f}$ and *Vangl2*$^{f/f}$*Ltf*$^{Cre/+}$ mice (n = 3 independent experiments). Arrows indicate locations of glands. Asterisks indicate embryos. Scale bar: 500 μm. **c,** Co-IP shows FOXA2 interacts with ESR1 (n = 3 independent experiments). Arrow indicates FOXA2 band. WB: Western blot.

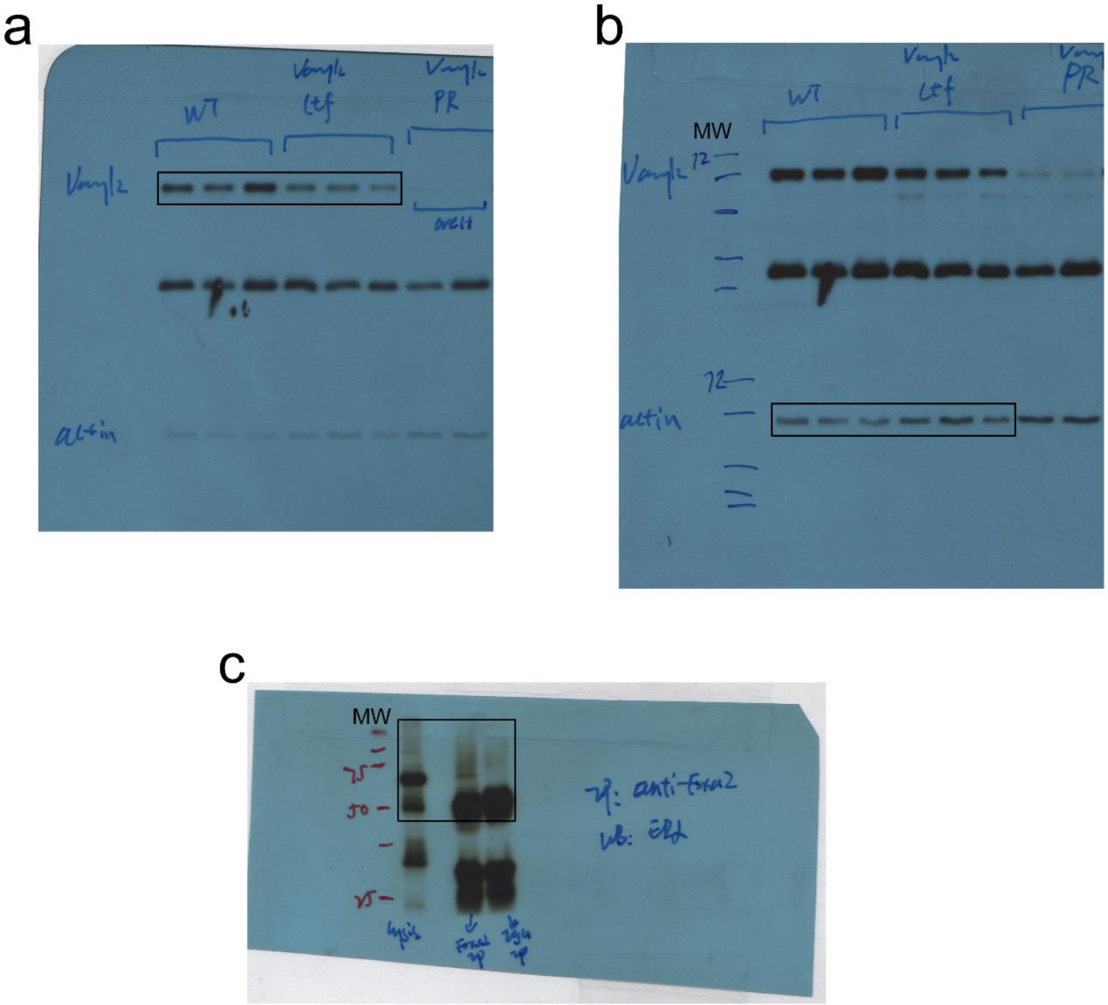

Supplementary Figure 7

Supplementary Figure 7 | Scans of the uncropped films for Western Blotting. **a** and **b** show the different exposure times for the same blot for Supplementary Figure 1f. **c**, depicts the uncropped scan of the film for Supplementary Figure 6c. MW, molecular weight.
