# Supplementary material for: Tridimensional visualization reveals direct communication between the embryo and glands critical for implantation
Source: Nat Commun. 2018 Feb 9;9:603. doi: 10.1038/s41467-018-03092-4 (PMC5807548; doi:10.1038/s41467-018-03092-4)
Supplement: Supplementary file 3 — Description of Additional Supplementary Files [file 41467_2018_3092_MOESM3_ESM.pdf]

Description of Additional Supplementary Files

File Name: **Supplementary Movie 1**

Description: **Movie of 3D imaging of a Day 5 implantation site in *Rosa26*^{tdTomato}*Ltf*$^{Cre/+}$reporter mice showing topography of glands-crypt-embryo interplay (Related to Fig. 3b).** The lumen and glands were segmented and marked with different pseudo-colours. Note the emergent glands from the crypt. *, embryo.

File Name: **Supplementary Movie 2**

Description: **Whole mount staining of CDH1 in day 5 *Vangl2*$^{f/f}$*Ltf*$^{Cre/+}$ uterus (Related to Fig. 3d).** The immunostained tissue was segmented and marked with different pseudo-colours.

File Name: **Supplementary Movie 3**

Description: **Whole mount staining of CDH1 at implantation site in day 6 *Vangl2*$^{f/f}$ uterus (Related to Fig.5 d-f).** The immunostained tissue was segmented into lumen and glands by different pseudo-colours. The glands at the implantation site were further extended and stretched due to growing decidua.

File Name: **Supplementary Movie 4**

Description: **Whole mount immunostaining of CDH1 of the implantation site in day 6 *Vangl2*$^{f/f}$*Ltf*$^{Cre/+}$ uterus (Related to Fig. 5 g-i).** The immunostained tissue was segmented into lumen and glands and decorated with pseudo-colours. The crypt is small and glands at the implantation site are less extended.

File Name: **Supplementary Movie 5**

Description: **3D display of D8 implantation site stained with CDH1 in *Vangl2*$^{f/f}$ uterus (Related to Fig. 8 a-c).** The glands are pushed into undifferentiated decidual stromal cells due to the expanded decidua and growing embryo. Note that many glands are separated from the crypt and nearby lumen. The immunostained tissue was segmented into lumen, glands and embryo and decorated with pseudo-colours.

File Name: **Supplementary Movie 6**

Description: **3D display of D8 implantation site immunostained with CDH1 in *Vangl2*$^{f/f}$*Ltf*$^{Cre/+}$ uterus (Related to Fig. 8 d-f).** Most glands are intact as the embryo growth is halted with a roundish and small embryo. The immunostained tissue was segmented into lumen, glands and embryo and decorated with pseudo-colours.
